# Family sports intergenerational influence effect and related factors analysis in the Yangtze Delta of China

**DOI:** 10.3389/fpsyg.2025.1592844

**Published:** 2025-08-07

**Authors:** Kun Yang, Wenduo Liu, Zhengxue Song

**Affiliations:** ^1^Liaoning Institute of Basic Medical Sciences, Shenyang, China; ^2^Department of Sports Science, College of Natural Science, Jeonbuk National University, Jeonju, Republic of Korea; ^3^College of Sports Science, Shenyang Normal University, Shenyang, China

**Keywords:** intergenerational influence, family study, sports behavior, sports motivation, sports condition

## Abstract

**Background:**

The intergenerational influence of family sports behavior is not only related to the interaction and imitation between the two generations, but also to factors such as family culture and socioeconomic status.

**Objective:**

This study aims to investigate the intergenerational influence and determinants of family physical activity behavior in the Yangtze River Delta region of China.

**Methods:**

This study investigated sports motivation, sports conditions, and sports rating through parent–child scales distributed to 288 families with school-age children aged 9–19 in the same area. Information on the gender and age of the children in each family, as well as the age, occupation, income, and educational background of the parents, was collected. Finally, the above results were statistically analyzed using the t-test and One-way analysis of variance.

**Results:**

The results show that caregiving parents’ gender has a significant impact on offspring sports motivation (OSM). The differences in the occupations of their parents have a significant impact on OSM, Offspring Sports Rating (OSR), and Offspring Sports Condition (OSC), and the sports-related occupations parents have a significant positive effect on the development of their offspring’s sports behavior. As well as differences in the educational background of their parents have a significant impact on OSC. On the other hand, the offspring’s influence on parental sports motivation (PSM) varies at different ages.

**Conclusion:**

Parental gender, education, occupation and children’s age are the key factors in the intergenerational influence of family sports behavior in the Yangtze River Delta region of China.

## 1 Introduction

Children and parents spend the most time at home together, and the high level of trust between them lays the foundation for the influence of sporting behavior. Parents had a profound influence on their children’s sporting development through the multidimensional approach (Dagkas and Burrows, 2016; Knight et al., 2016; Wuerth et al., 2004). Moreover, parents are the initiators of their children’s participation in sports and the central providers of the opportunities and resources needed during the upward phase of their sporting careers (Harwood and Knight, 2009; Novak et al., 2020). With the rise of health awareness, more and more parents see sports to promote their children’s physical and mental health development, rather than as a career path (Knoester and Allison, 2022). It is undeniable that parents play an important role in promoting children’s participation in sports, and their influence runs through the entire chain of motivating their children to participate in sports, providing them with resources, and shaping their results. It is worth noting that family behavior is formed through a two-way dynamic interaction between parents and children (Mead, 1970). The influence of parents is only one aspect of the family influence mechanism; the reverse influence of children on their parents’ behavior should not be overlooked. Given the universality and inherent complexity of this dynamic interactive process, it is crucial to identify the key factors influencing family exercise behavior in intergenerational interaction patterns.

Mead first proposed an intergenerational theory of family behavior in 1970, and believed that this intergenerational influence was mutual (Mead, 1970), but his theory did not specifically explore the intergenerational effects of family physical activity. As society’s concern about adolescent health issues heats up, the “intergenerational transmission effect” of family sports has gradually become a research hotspot. For example, the first coach of a professional athlete is the parents, the parents influence their children’s motivation to exercise through their behavior, and the availability of sports resources in the home significantly affects children’s sports conditions (Hayoz et al., 2019; White et al., 2023). Although the literature has gradually revealed the phenomenon of intergenerational influence on family sports behavior, the following key issues have not been fully explored. Such as the existing literature mostly treats parents as a homogeneous group, without distinguishing the differences in the intergenerational influence of fathers and mothers on their children’s family sports behaviors. The literature that homogenizes family situations does not adequately discuss the intergenerational influence of differences in parents’ educational background, income level, occupation, and age. Mead emphasized the “two-way nature” of intergenerational influence, that is the influence of parents on children and the influence of children on parents occurs at the same time. This is often missing in the current literature on the intergenerational influence of sports behavior. In addition, western research dominates the current theoretical construction, and the concept of “academic qualifications first” common in east Asian families may weaken the intensity of the intergenerational influence of sports behavior in the family. Especially, the unique one-child policy in China and the process of urbanization may have a combined effect, and the intergenerational influence of sports behavior may differ from western research.

Within the framework of intergenerational influence, the two-way interactive mechanism of family sports behavior is mainly shaped by three key factors: intergenerational influence on sports rating (frequency, duration, and intensity), intergenerational influence on sports motivation, and intergenerational influence on sports conditions. This framework provides a theoretical basis for identifying the intergenerational effects of family sports behavior.

Current research indicates that there is a significant intergenerational influence on children’s sport ratings. Specifically, parents significantly increased their children’s athletic performance compared to other families by spending more time accompanying their children in sports (Kovács et al., 2024). Parents with high levels of sports activity tend to incorporate sport into family life, viewing it as part of daily life rather than a specific task. This phenomenon also significantly affects their children’s sports activity levels (Fredricks and Eccles, 2005). Parents with high levels of exercise intensity also have children who exhibit higher levels of exercise intensity in their daily training (Lau et al., 2016).

In terms of family motivation for sport, parents promote their children’s motivation to exercise by watching sports competitions together, sharing their sports experiences, or participating in parent–child sports activities (Liu et al., 2023). In particular, parents who are enthusiastic about demonstrating sports techniques have a significant effect on motivating their children to exercise (Bunds et al., 2018).

In terms of family conditions for sport, children’s participation in sports usually requires parents to provide the necessary conditions to support it. This includes, but is not limited to, the time and expenses required to transport children to training venues, payment of sports club fees, and restrictions on taking time off work to accompany children to competitions, etc. (Lauer et al., 2010).

Yangtze River Delta, as a typical region with active economy and fierce competition in education, may reflect the typical characteristics of the exercise patterns of Chinese families. Therefore, this study targeted families with primary, middle and high school students in representative cities in the Yangtze River Delta region and conducted a scale survey to explore the intergenerational influence and determinants of family sports behavior in the region. This study will provide a strong reference for early childhood family sports behavior intervention, increasing the sports and healthy population, and exploring the fields of family sports products and services.

## 2 Materials and methods

### 2.1 Research design and process

**Figure 1:**
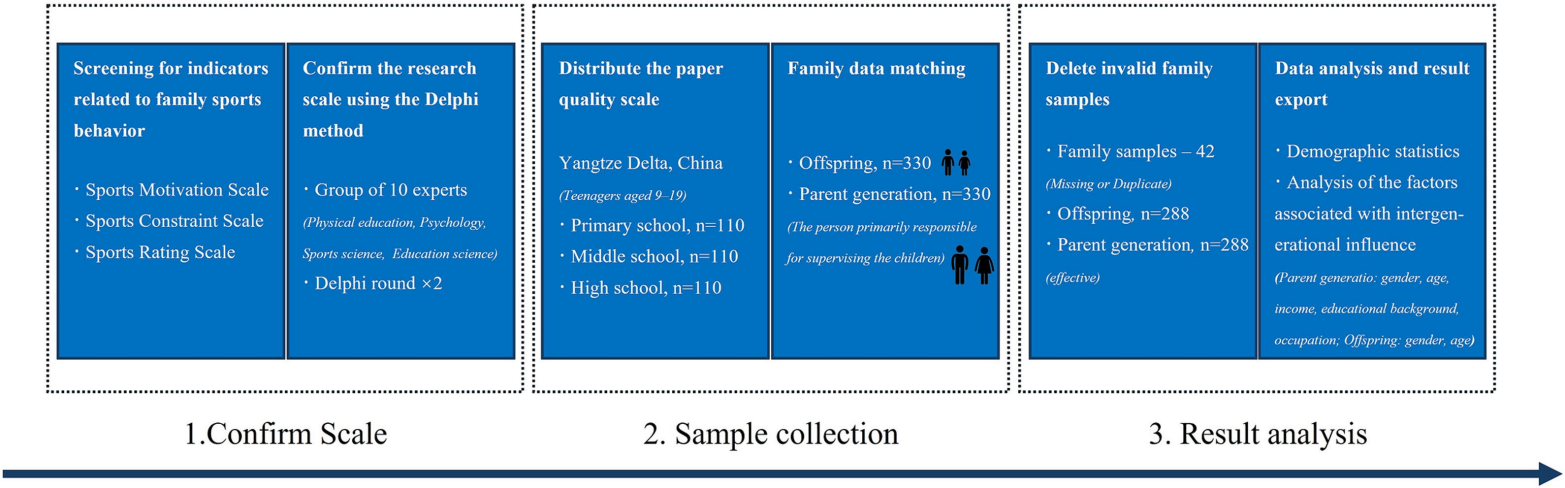
Research design and process.

The design and process of this study are shown in Figure 1. First, relevant indicators were selected based on previous research, and the research scale was confirmed using the Delphi method. Then, the scale was printed and distributed in three schools (one each for primary school, middle school, and high school) in City N of the Yangtze Delta. The subjects were explained how to use the scale, and the children took the scale home to complete the parent and child scales together as homework with their parents. Finally, the researcher will collect the scales, delete the scales with duplicate options and missing information, and enter the data, conduct statistical analysis, and export the results for the valid data that have been finally completed.

### 2.2 Research tools and testing

The measurement tools used in this study were based on the Sports Motivation Scale (Mallett et al., 2007; Pelletier et al., 1995), the Physical Activity Rating Scale (Hashimoto, 1990), and the Leisure Constraint Scale (Crawford and Godbey, 1987; Crawford et al., 1991), with modifications and additions. The scale used the Likert-5 point scale, with 5 being strongly agree and 1 being strongly disagree (Malik et al., 2021). As these scales were created by native English speakers, the items in the scales were optimized locally using the Delphi method (Landeta, 2006) to enhance their suitability for use in China.

The Delphi method expert group consists of 10 Chinese practitioners in related fields (Supplementary Table 1), including physical education teachers from high schools, middle schools and primary schools, and professors of psychology, physical education and Sports science at universities. According to the rules of the Delphi method, the researchers sent the draft scale to the online mailbox of the experts by e-mail. After the evaluation was completed, the results were collected and adjusted accordingly before being sent for review again. The standard for scoring by experts is 1 (disagree strongly) to 5 (agree strongly). The measurement standard is that if the average value (mean) is >3.5 and the coefficient of variation (Cv) is <0.25, then the standard is met and the entry proceeds to the next round of verification. If the standard is not met, the entry will be deleted. Based on the feedback from the first round of expert opinion, the researchers deleted two items with an average score below 3.5, which were considered unimportant, and revised the wording of eight items. The expert opinion was to revise the wording of some items to make them easier for the offspring to understand and facilitate answering. After revising the scale, the researchers organized a second round of expert scoring. In the second round of expert scoring, the mean value and Cv of all items met the numerical standards, and no other modifications were proposed. Therefore, a third round of expert testing was not conducted. Supplementary Table 2 shows the content of the scale and the results of the second round of expert scoring.

Although the Delphi method is effective in assessing content validity, it cannot assess the structural validity of the scale. In this study, we used principal component analysis (PCA), exploratory factor analysis (EFA) and confirmatory factor analysis (CFA) methods to comprehensively assess the potential relationships between variables and data structures. The results are shown in the Supplementary Tables 3, 4. Based on the results of the structural validity analysis, measurement items with factor loadings below 0.6 (Family sports condition: FSC3) were eliminated according to the standards of the research design, enhancing the structural validity and robustness of the model.

A correlation test was performed on the results of the collated data to confirm the existence of mutual influence between parent–child behaviors to ensure the validity of the main results of this study. The results are shown in the Supplementary Table 5.

### 2.3 Participants and scales collection

Power analysis (G power 3.1, Germany) showed that a minimum of 60 participants were required to achieve a minimum efficacy of 0.8 in this study. At the same time, this study adopted the recommendations of the expert group and distributed family activity scales to three types of families, with 220 scales distributed to each type of family (including 110 scales distributed to primary school students and their parents, 110 scales distributed to middle school students and their parents, and 110 scales distributed to high school students and their parents), for a total of 660 participants. In China, primary school students are typically between the ages of 6 and 12, but in actual surveys, children between the ages of 6 and 8 have difficulty understanding words and are unable to complete the scales independently even with guidance from their parents. Therefore, this study excluded families with students aged 6 to 8.

Therefore, the sample of children attending primary school was revised to 9–12 years old. The middle school sample corresponded to the 13–15 years old child sample, and the senior high school sample corresponded to the 16–19 years old child sample. For the selection of the parent sample, only one parent who was responsible for the daily care and supervision of the child was surveyed in this study. All parent samples were not specially screened and were randomly generated from the actual family situations of the child samples. Each participant voluntarily agreed to participate in the study and signed a written informed consent. This project was reviewed and approved by the Institutional Review Board.

The survey scale was printed on traditional paper. The scale was bound in duplicate as a set of parent–child scales, and the scales were same. After obtaining authorization from the school administrators and the participating families, the researchers distributed the scales in each school. The researchers informed the students on site that they were responsible for filling in the child part of the scale, while the parents filled in the parent part, and ensured that the data of the parents and children in the same family must match.

After collecting the questionnaires, 84 scales (42 from parents and 42 from children) were found to be invalid (duplicate or missing information) and could not be analyzed. Ultimately, 576 family activity scales (288 from parents and 288 from children) were used for the analysis of results. The specific demographic data is shown in the Table 1.

**Table 1:** Demographic characteristics of family members.

Variable	Content	Parent (*N* = 288)	Offspring (*N* = 288)
Gender (*N* = 576)	Male	151 (52.4%)	146 (51%)
	Female	137 (47.6%)	142 (49%)
Age (*N* = 576)	9–12		94 (33%)
	13–15		94 (33%)
	16–19		100 (34%)
	20–29	3 (1%)	
	30–39	107 (37%)	
	40–49	160 (56%)	
	50–59	12 (4%)	
	60–69	6 (2%)	
Education (*N* = 288)	High school or below	108 (37%)	
	Junior college	79 (27%)	
	Bachelor	71 (25%)	
	Master	19 (7%)	
	Doctor	11 (4%)	
Income (*N* = 288)	≤345USD (2,500 CNY)	9 (3%)	
	345–553USD (2501–4,000 CNY)	51 (18%)	
	553–691USD (4001–5,000 CNY)	41 (14%)	
	691–829 USD (5001–6,000 CNY)	41 (14%)	
	≥829USD (6001CNY)	146 (51%)	
Occupation (*N* = 288)	Sports-related Practitioners	10 (3.5%)	
	Civil Servants	39 (13.5%)	
	Corporate Executive	55 (19.1%)	
	Company Employee	64 (22.2%)	
	Freelancer	111 (38.5%)	
	Unemployed	9 (3.1%)	

### 2.4 Data analysis

The data was expressed as the mean ± Standard Deviation (SD). Descriptive statistics were used for the demographic variables. Independent sample *t*-test or One-way ANOVA analysis of variance are used to analyze the influencing factors in relation to the intergenerational effect. The Tukey *post hoc* test was used to determine significance. The significance level was set at *p* < 0.05. The statistical analysis was performed using SPSS 27.0 (Inc., Chicago, IL, United States).

## 3 Results

### 3.1 Demographic data

A total of 330 parent–child scales were distributed for this study. After eliminating 42 duplicate scales and scales with missing information, 288 scales were used for the results analysis. There are 137 female parents, 151 male parents, 146 male offspring, and 142 female offspring (Table 1). The gender of the sample is evenly distributed, which meets the requirements of the research. The specific demographic data is shown in the Table 1.

### 3.2 Differences in the influence of the parent generation on the sports behavior of their offspring

**Figure 2:**
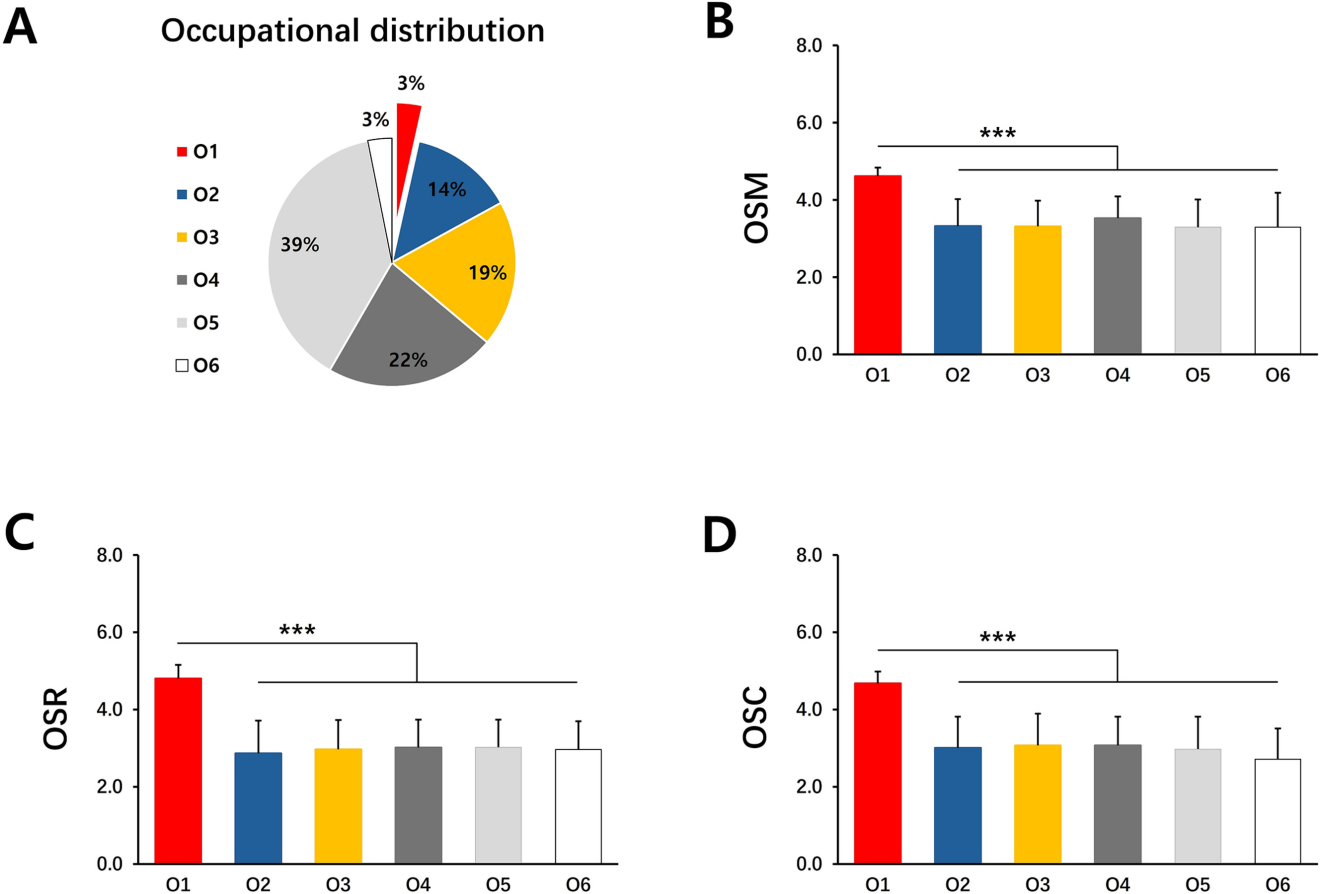
The influence of the occupation of the parent generation on the sport behavior of the offspring. **(A)** Occupational distribution of parent generation. **(B)** The influence of parents’ occupational differences on OSM. **(C)** The influence of parents’ occupational differences on OSR. **(D)** The influence of parents’ occupational differences on OSC. O1, Sports-related Practitioners; O2, Civil Servants; O3, Corporate Executive; O4, Company employee O5, Freelancer; O6, unemployed. Mean ± SD. *p < 0.05, **p < 0.01, ***p < 0.001.

To illustrate the influence of parental factors on the sports behavior of offspring, we analyzed the influence of parental gender, age, education, income, and occupation on Offspring Sports Motivation (OSM), Offspring Sports Rating (OSR), and Offspring Sports Condition (OSC). As shown in the results of Table 2, although there is no significant difference in the impact of the gender of the parent generation who is responsible for childcare and supervision on OSC and OSR, but the gender difference has a significant impact on OSM (Table 2). The impact of male on OSM is significantly higher than that of female (Table 2). The results of the parents’ age (Supplementary Table 6) and income (Supplementary Table 7) did not show a significant impact on the sports behavior of their offspring. However, the difference in parents’ occupation (Table 3) and education (Table 4) has a significant impact on their children’s sports behavior. Differences in the occupations of the parents had a significant impact on OSM, OSR and OSC (Table 3). In the results of this survey, although parents who are sports-related practitioners only account for 3% of the total (Figure 2A), this group shows a significant influence on the sports behavior of future generations. The offspring of sports-related practitioners have significantly higher OSM, OSR and OSC than the offspring of other professions (Figures 2B–D). The difference in parental education experience also reflects the impact on the offspring’s OSC, although the results of the Tukey post-hoc test do not reflect the significant advantages of any education level, but the data shows a trend of increasing OSC scores with increasing parental education (Table 4). In short, gender, occupational and education differences between parents are the main factors affecting their children’s sports behavior.

**Table 2:** The influence of caregiving parents’ gender on the sports behavior of the offspring.

Variable	Men	Women	*p*
OSM^*^	3.50 ± 0.68	3.28 ± 0.72	0.011
OSR	3.11 ± 0.82	2.97 ± 0.77	0.130
OSC	3.14 ± 0.84	2.98 ± 0.85	0.132

Mean ± SD; The *p*-value is from an independent sample *t*-test; *p < 0.05.

**Table 3:** The influence of parental occupations on the sports behavior of the offspring.

Variable	O1	O2	O3	O4	O5	O6	*p*
OSM^***^	4.61 ± 0.22	3.32 ± 0.69	3.31 ± 0.66	3.52 ± 0.56	3.29 ± 0.72	3.29 ± 0.88	0.000
OSR^***^	4.80 ± 0.35	2.80 ± 0.85	2.96 ± 0.75	3.01 ± 0.72	3.02 ± 0.71	2.96 ± 0.73	0.000
OSC^***^	4.68 ± 0.30	3.00 ± 0.81	3.07 ± 0.81	3.07 ± 0.74	2.97 ± 0.84	2.71 ± 0.80	0.000

O1, Sports-related Practitioners; O2, Civil Servants; O3, Corporate Executive; O4, Company employee O5, Freelancer; O6, unemployed; Mean ± SD. The *p*-value is from One-way ANOVA; ***p < 0.001.

**Table 4:** The influence of parental education on the sports behavior of the offspring.

Variable	High school and below	Junior college	Bachelor	Master	PhD	*p*
OSM	3.31 ± 0.78	3.45 ± 0.69	3.37 ± 0.68	3.62 ± 0.36	3.59 ± 0.60	0.315
OSR	2.95 ± 0.81	3.17 ± 0.82	2.99 ± 0.82	3.14 ± 0.47	3.21 ± 0.82	0.355
OSC^*^	2.87 ± 0.85	3.18 ± 0.90	3.10 ± 0.77	3.35 ± 0.64	3.38 ± 0.91	0.028

Mean ± SD; the *p*-value is from One-way ANOVA; *p < 0.05.

### 3.3 Differences in the influence of the offspring on the sports behavior of their parent generation

**Figure 3:**
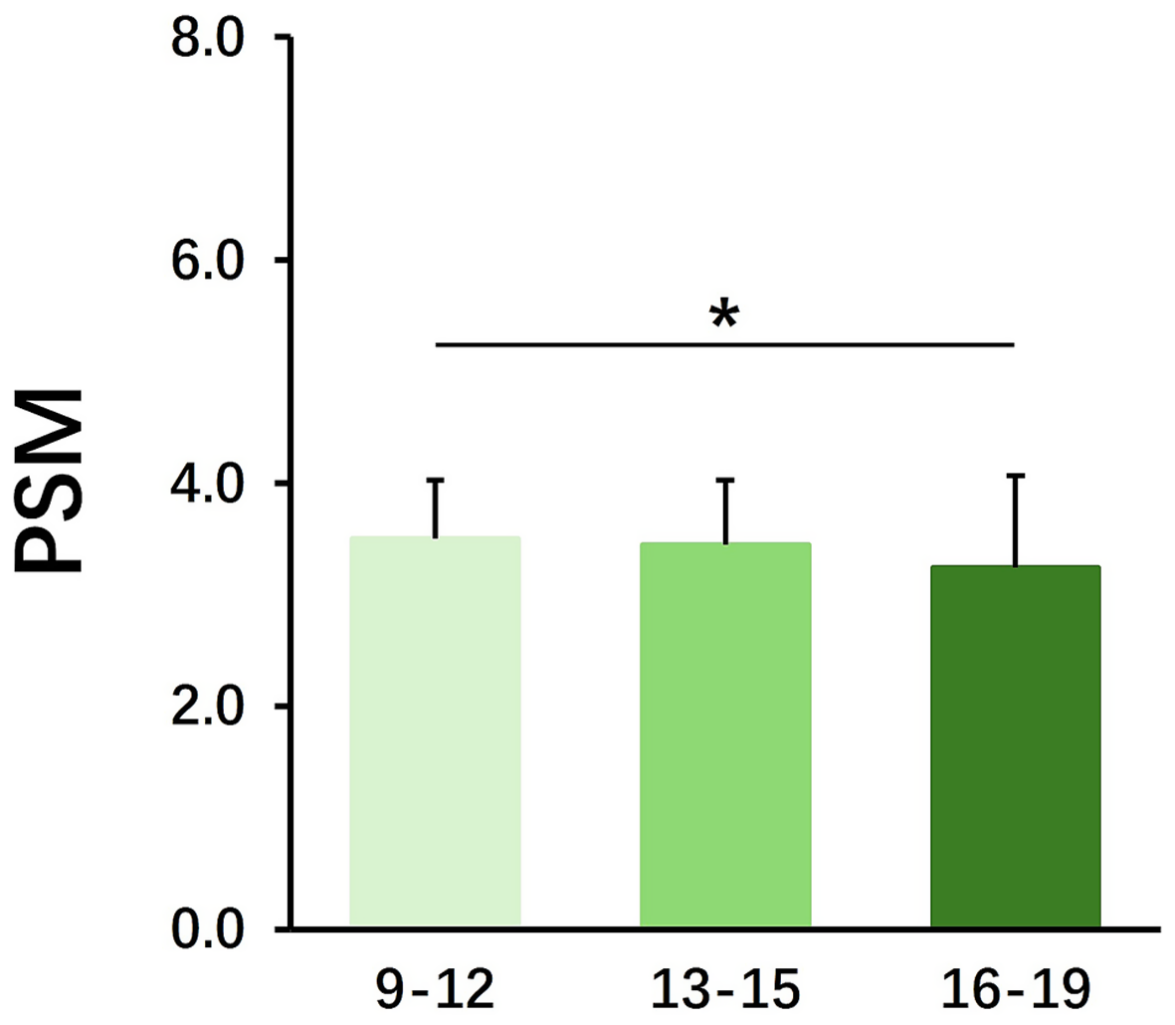
The effect of the age of the offspring on PSM. Mean ± SD; *p < 0.05, **p < 0.01, ***p < 0.001.

To illustrate the influence of the offspring factor on the sports behavior of the parents, we analyzed the influence of the offspring’s gender and age on Parental Sports Motivation (PSM), Parental Sports Rating (PSR) and Parental Sports Condition (PSC). The results show that differences in the gender of the offspring do not have an additional effect on the sports behavior of the parents (Table 5). However, the age difference of the offspring has a significant effect on the PSM (Table 6). The effect of offspring aged 9–12 on the PSM is significantly higher than that of offspring aged 16–19, while there is no significant difference between offspring aged 13–15 and the remaining age groups (Figure 3). The above results show that the age difference between the offspring is the main factor affecting parents’ sports behavior.

**Table 5:** The influence of offspring gender on the sports behavior of the parent generation.

Variable	Man	Women	*p*
PSM	3.37 ± 0.71	3.42 ± 0.64	0.510
PSR	2.96 ± 0.80	3.01 ± 0.78	0.570
PSC	3.10 ± 0.84	2.97 ± 0.84	0.171

Mean ± SD; the *p*-value is from an independent sample *t*-test.

**Table 6:** The influence of offspring age on the sport behavior of the parent generation.

Variable	AGE 9–12	AGE 13–15	AGE 16–19	*p*
PSM^*^	3.50 ± 0.53	3.45 ± 0.58	3.24 ± 0.83	0.019
PSR	2.93 ± 0.68	3.12 ± 0.68	2.91 ± 0.94	0.131
PSC	3.12 ± 0.84	3.04 ± 0.72	2.95 ± 0.94	0.403

Mean ± SD; the *p*-value is from One-way ANOVA; *p < 0.05.

## 4 Discussion

The results of this study show that there is a clear intergenerational effect on the sports behavior of student families in the Yangtze River Delta region of China. Among them, the caregiving parents’ gender, occupational as well as education differences, have a significant impact on their children’s sports behavior (Tables 2–4). In terms of gender, male caregivers are more conducive to the development of children’s sports behavior, and practitioners (Table 2). People in the occupational category of sports-related occupations also show a promotion of the sports behavior of the next generation (Figure 2). In contrast, the factors influence of the offspring on the parents are mainly related to age (Table 6). Children aged 9–12 are more likely to influence their parents’ sports activity (Figure 3).

The results of the effect of parent generation gender differences on OSM found that the effect of males was higher than that of females (Table 2), indicating that fathers may provide more sports motivation for their children. The findings of Fredricks and Eccles (2002) also show that fathers provide more motivation for sports to their children than mothers do in middle-class American families with school-age children (grades 1–12, *N* = 514, 50% male, 50% female), which partially supports the results of this study (Fredricks and Eccles, 2002). The age, education, gender ratio, and number of offspring in the aforementioned previous studies and the economic level of the parents in this study sample highly overlap. Although the family income collected in this study only refers to one of the parents, 36% of parents have a university degree or higher (Table 1), 51% have a monthly income of more than 829 USD (Table 1), which is already at the level of the Chinese middle class (Bonnefond and Clément, 2014). Based on Fredricks’ findings, the phenomenon that fathers play a greater role in providing motivation for sports may not be limited to specific countries (such as the United States), but also exists in the Chinese family sample represented in this study.

Although the sex difference of the parent generation affects OSM, it has no effect on OSC and OSR (Table 2). The results of the Hayoz et al. (2019) study also partially support the results of this study, as OSC and OSR did not differ according to the gender of the parents (Hayoz et al., 2019). From the perspective of family sports behavior, OSR, as an indicator of children’s actual amount of sports (the most important indicator), did not appear to have an impact (Table 2). Based on the data from this study, no significant effect of parental gender on children’s actual physical activity levels was found.

This study found that differences in parents’ occupations have a significant impact on the development of their children’s OSM, OSR and OSC, and that sports-related occupations have a significant advantage over all other occupations in influencing their children’s sports behavior. However, no difference was found between the remaining occupations (Figure 2). Zaccagni et al. (2017) found that differences in parents’ occupations had an impact on their children’s (*N* = 2,682, 49.5% girls, 50.5% boys, aged 5.9 +/− 0.3, Italian) motivation, amount, and support for sports (Zaccagni et al., 2017). Although the specific occupations of the parents were not explained, the conclusions were consistent with this study. Sports practitioners typically have strong sports expertise, sports skills, and industry understanding (Graham et al., 2016). Parents’ daily participation in and guidance of sports can set an example for their children, stimulate their interest and motivation, and enhance their motivation to sports (Harrington, 2006). Sports practitioners parents whose jobs provide access to sports facilities, training resources, and professional guidance have a professional advantage (Ede et al., 2012). It is more conducive to allowing children to engage in physical activities in a safe and healthy environment, greatly reducing the risk of sports. Due to the high frequency of parents’ participation in daily sports, children are more likely to be exposed to sports in their daily lives, and under the guidance of their fathers, the frequency, duration, and quality of participation are often higher (Tamminen and Holt, 2012). Compared with other occupations, sports families may have unique advantages in promoting their children’s motivation to participate in sports, level of support for sports, and actual level of physical activity.

In this study, 36% of parents have a university degree or higher (Table 1). The educational level of parents has a positive effect on OSC (Table 4). Stalsberg and Pedersen (2010) believe that a high level of education leads to high income, which in turn enables children to obtain high-level sports conditions. In contrast to Stalsberg’s findings, no phenomenon of family income background influencing children’s access to sports was observed in the samples from China’s Yangtze River Delta region.

The influence offspring of age differences in PSM shows that children aged 9–12 have a greater influence on PSM than those aged 16–19 (Figure 3). This is an interesting finding, as children’s influence on their parents’ sports behavior diminishes as they grow older, and their personal abilities increase. As children grow and mature, and as psychological changes occur during adolescence, the amount of time parents spend engaging in joint activities with their children decreases significantly over time (Dubas and Gerris, 2002).

The sample for this study came from the Yangtze River Delta region of China. This region is one of the key areas of economic development in China. Although the sample was not specifically selected, 51% of the parents surveyed had middle-class household incomes, which may limit the generalizability of the results. In addition, this study relied on the self-assessment of respondents in the scale, which may lead to social desirability bias or recall bias. Moreover, follow-up studies can combine self-reported behavior with actual movement data to verify research results and reduce bias. Future research can use a longitudinal design to track changes in family sports behavior and focus on long-term changes in sports behavior under intergenerational relationships. It is also possible to consider expanding the sample size to enhance the representativeness of the study.

## 5 Conclusion

The results of this study indicate that gender, education and occupational differences between parents are the main factors affecting their children’s sports behavior. As well as the age difference between the offspring is the main factor affecting parents’ sports behavior. In the future, in-depth discussions should be held on key factors such as the choice of sports, intergenerational common sports behaviors, and the intergenerational impact of sports consumption concepts, to provide a more powerful reference for the development of the regional sports industry and increase the sports population.

## Data Availability

The raw data supporting the conclusions of this article will be made available by the authors, without undue reservation.
