# Supplementary material for: Family sports intergenerational influence effect and related factors analysis in the Yangtze Delta of China
Source: Front Psychol. 2025 Aug 7;16:1592844. doi: 10.3389/fpsyg.2025.1592844 (PMC12368973; doi:10.3389/fpsyg.2025.1592844)
Supplement: Supplementary file 1 [file Table_1.docx]

Supplementary Material

**Supplementary Table 1. Expert information statistics.**

| **Experts name** | **Field** | **Identity** | **Working place** |
| --- | --- | --- | --- |
| Gu* | Physical education | Teacher | ***High school |
| Chen** | Physical education | Teacher | ***Junior high school |
| Zhou* | Physical education | Teacher | ***Junior high school |
| Wang* | Physical education | Teacher | ***Primary school |
| Wang** | Psychology | Associate professor | ****University |
| Zhang** | Psychology | Associate professor | ****University |
| Che* | Sports science | Associate professor | ****University |
| Liu* | Sports science | Professor | ****University |
| Xia** | Sports science | Professor | ****University |
| Zhang* | Education science | Professor | ****University |
| Zhang** | Education science | Associate professor | ****University |

**Supplementary Table 2. Experts' second round evaluation result.**

| **Index** | **Question** | **Mean** | **Cv** | **SD** |
| --- | --- | --- | --- | --- |
| **Family Sports Motivation** | **FSM1**: Sport improves body shape and has a body-shaping effect (body shape improvement). | 4.9 | 0.07 | 0.33 |
|  | **FSM2**: Training and competition can explore high-level sports skills and feel happy (exploration of skills). | 4.3 | 0.20 | 0.87 |
|  | **FSM3**: Enjoys praise from others when exercising (external motivation). | 4.1 | 0.23 | 0.93 |
|  | **FSM4**: Sports relax me and improve my mood (emotion regulation). | 4.8 | 0.09 | 0.44 |
|  | **FSM5**: Sports can improve physical fitness and prevent disease (health  promotion). | 4.6 | 0.16 | 0.73 |
|  | **FSM6**: I feel a sense of accomplishment when I win in a competition (accomplishment). | 4.4 | 0.16 | 0.73 |
| **Family sports condition** | **FSC1**: I feel comfortable in sports facilities (environment and equipment). | 4.8 | 0.09 | 0.44 |
|  | **FSC2**: I have professional coaching (professional guidance). | 4.1 | 0.23 | 0.93 |
|  | **FSC3**: My physical condition is suitable for exercise (health condition). | 3.8 | 0.22 | 0.83 |
|  | **FSC4**: Family support my sport (family support). | 4.6 | 0.19 | 0.88 |
|  | **FSC5**: The sports venue is easily accessible (transport accessibility). | 4.9 | 0.07 | 0.33 |
|  | **FSC6**: I know where to get sports resources (resource acquisition). | 4.6 | 0.16 | 0.73 |
| **Family Sports Rating** | **FSR1**: Duration of each exercise (under 10 minutes; 11–20 minutes; 21–30 minutes; 31–40 minutes; over 41 minutes) | 4.9 | 0.07 | 0.33 |
|  | **FSR2**: Frequency of exercise per week (1; 2; 3; 4; 5 more times). | 4.9 | 0.07 | 0.33 |
|  | **FSR3**: Intensity of each exercise (Very easy, no sweating, breathing is even; Slightly laborious, slightly sweating, breathing is quickened, can talk normally; Requires some effort, sweating is obvious, breathing is quickened, having a conversation is a bit difficult; Noticeable laborious, sweating profusely all over, breathing is quickened, unable to have an easy conversation; Almost reaching the limit of exercise, unable to last long, breathing is difficult, unable to have a conversation). | 4.8 | 0.14 | 0.67 |

| **Variable** | **Factor**  **Loadings** | **Cronbach’s**  ***ɑ*** | **Percentage of variance** |
| --- | --- | --- | --- |
| **FSM1** | .845 | .932 | 31.590% |
| **FSM2** | .754 |  |  |
| **FSM3** | .748 |  |  |
| **FSM4** | .796 |  |  |
| **FSM5** | .791 |  |  |
| **FSM6** | .783 |  |  |
| **FSC1** | .772 | .878 | 23.974% |
| **FSC2** | .643 |  |  |
| **FSC4** | .801 |  |  |
| **FSC5** | .851 |  |  |
| **FSC6** | .629 |  |  |
| **FSR1** | .843 | .872 | 18.314% |
| **FSR2** | .803 |  |  |
| **FSR3** | .814 |  |  |

**Supplementary Table 3. Data validity check.**

Extraction Method: Exploratory factor analysis (EFA), Principal Component Analysis (PCA), Cronbach's Alpha Reliability Coefficient and KMO=0.928; n=576 (Parent 288+ Offspring 288); Family sports motivation (FSM), Family sports conditions (FSC), Family sports rates (FSR).

**Supplementary Table 4. Measurement model evaluation.**

|  | **Composite Reliability** | **AVE** | **x^2^/df** | **x^2^** | **RMSEA** | **SRMR** | **GFI** | **CFI** | **NFI** | **TLI** |
| --- | --- | --- | --- | --- | --- | --- | --- | --- | --- | --- |
| FSM | .934 | .701 | 2.059 | 152.382 | .061 | .032 | .930 | .973 | .949 | .967 |
| FSC | .893 | .627 |  |  |  |  |  |  |  |  |
| FSR | .873 | .699 |  |  |  |  |  |  |  |  |

Extraction Method: Confirmatory factor analysis (CFA); n=576 (Parent 288+ Offspring 288); Family sports motivation (FSM), Family sports conditions (FSC), Family sports rates (FSR).

**Supplementary Table 5. Correlation analysis of intergenerational influence of family sports behavior.**

| **Variable** | **PSM** | **PSR** | **PSC** | **OSM** | **OSR** | **OSC** |
| --- | --- | --- | --- | --- | --- | --- |
| **PSM** | 1.000 |  |  |  |  |  |
| **PSR** | 0.595^**^ | 1.000 |  |  |  |  |
| **PSC** | 0.675^**^ | 0.520^**^ | 1.000 |  |  |  |
| **OSM** | 0.390^**^ | 0.376^**^ | 0.280^**^ | 1.000 |  |  |
| **OSR** | 0.369^**^ | 0.432^**^ | 0.222^**^ | 0.630^**^ | 1.000 |  |
| **OSC** | 0.325^**^ | 0.297^**^ | 0.302^**^ | 0.650^**^ | 0.551^**^ | 1.000 |

*The correlation is significant at 0.05; **The correlation is significant at 0.01 (two-tailed).

**Supplementary Table 6. The influence of the age of the parent generation on the sports behavior of the offspring.**

| **Variable** | **Age:**  **20-29** | **Age:**  **30-39** | **Age:**  **40-49** | **Age:**  **50-59** | **Age:**  **60-69** | ***p*** |
| --- | --- | --- | --- | --- | --- | --- |
| **OSM** | 4.00±0.44 | 3.40±0.62 | 3.40±0.76 | 3.11±0.81 | 3.50±0.40 | 0.375 |
| **OSR** | 3.77±1.17 | 3.09±0.71 | 2.98±0.85 | 3.11±0.88 | 3.27±0.44 | 0.372 |
| **OSC** | 3.72±1.10 | 3.03±0.89 | 3.11±0.81 | 2.9±0.97 | 2.50±0.41 | 0.227 |

Mean ± SD; The *p*-value is from One-way ANOVA; * *p*＜0.05; ** *p*＜0.01; *** *p*＜0.001.

**Supplementary Table 7. The influence of the income of the parent generation on the sports behavior of the offspring.**

| **Variable** | **＜345USD *(2500CNY)*** | **345-553USD *(2500-4000CNY)*** | **553-691USD *(4001-5000CNY)*** | **691-829USD *(5001-6000CNY)*** | **＞829USD *(6000CNY)*** | ***p*** |
| --- | --- | --- | --- | --- | --- | --- |
| **OSM** | 3.12±0.97 | 3.41±0.78 | 3.44±0.69 | 3.29±0.80 | 3.42±0.64 | 0.641 |
| **OSR** | 3.11±0.79 | 3.09±0.92 | 3.15±0.83 | 2.99±0.73 | 3.01±0.77 | 0.854 |
| **OSC** | 2.64±0.94 | 3.03±0.88 | 3.13±0.91 | 3.09±0.40 | 3.08±0.79 | 0.624 |

Mean ± SD; The *p*-value is from One-way ANOVA; * *p*＜0.05; ** *p*＜0.01; *** *p*＜0.001.
